# The Texas Medical College and Hospital

**Published:** 1888-06

**Authors:** 


### EDITORIAL DEPARTMENT.

F. E. DANIEL, M. D., EDITOR AND PUBLISHER.

THIS JOURNAL, by unanimous vote of the members, was made the Official Organ of the Austin District Medical Society.

COLLABORATORS:

*Dr. R. M. Swearingen., Austin.*
*Dr. B. E. Hadra, Austin.*
*Dr. Geo. Cupples, San Antonio.*
*Dr. T. C. Osborn, Cleburne, Texas.*
*Dr E. J. Doering, Chicago.*
*Dr Odo Betz, Germany.*
*Dr. J. W. McLaughlin, Austin.*
*Dr. T. J. Tyner, Austin.*
*Dr. J. F. Y. Paine, Galveston.*
*Dr. E. Goldmann, San Jose, C*
*Dr. H. O. Marcy, Boston.*
*Dr. E. Meierhof, New York.*
*E. J. Beall, M. D., Fort Worth, Texas.*

### THE TEXAS MEDICAL COLLEGE AND HOSPITAL.

The successful inauguration of a School of Medicine in Texas is, justly, a cause for congratulation. It marks another important step in the wonderful progress which has characterized this giant young Republic,—and an era in the medical history of the State. It is a triumph over many, and serious obstacles.

This step has not been hasty, nor ill-advised; but is the outcome of serious and intelligent reflection and deliberation, and has been taken, in full knowledge and appreciation of the fact that medical education (so called) is too cheap,—and graduation too easy in the States; and that the country is overrun with poorly educated medical men, turned loose from schools inadequate to a thorough and proper training for the serious responsibilities of the calling. Texas is on record as calling for reform in medical teaching, and a higher grade of medical education. For years, her medical men, through their organization, have been knocking at the doors of the Legislature, asking that a law be passed, restricting the privilege of practicing Medicine, to the *qualified*,—the first step towards a higher educational standard on the part of Medical Colleges. She could poorly afford, then, to set in operation, a process for the further spread of the evil complained of; and does not propose to do so. The Texas Medical College is not to be but another added to the long list of second-rate schools.

The profession,—the people,—a due regard for the interests of the science of Medicine alike demand that it shall be as good as the best; and, in the course of a little while, if not at the very inception, it shall become second to none in America. In the very nature of things it must rank below certain old and well-established Schools for awhile; it were unreasonable to expect it to emerge, full-fledged,—as did Minerva from the brain of Jove; but there is so much of enterprise, so much of vitality in the germ, that its growth and development into a first-class School will be a matter of short time.

It is not proposed to make graduation easy, in order to attract a large class; that has been the bane and curse of the country,—but rather to make the instruction and the examinations so thorough as to give value and distinction to a diploma.

The Faculty, as far as chosen and announced, is a good one, all things considered, and as practitioners, have given evidence of sound education and ability in their several branches. But there are several branches which cannot be satisfactorily taught by the general practitioner, however able. The Chairs of Biology, Experimental Physiology and Pathology, and that of Chemistry, should be filled by the best teaching talent to be had anywhere; funds should be forthcoming for that, if for no other purpose; for, without facilities for thorough instruction in those important departments, the education imparted to the students will fall far short of what it should be. This, in our opinion, is one of the prime requisites of the School; and we are advised that steps are being taken to this end. Young, vigorous, and ambitious men, fresh from the clinics of New York, or London and Paris, trained and taught in all the latest phases of experimental reseach, and capable of imparting the sum of their knowledge, should be secured at whatever cost.

The Texas Medical College and Hospital is not the Medical Department of the University of Texas—as might be inferred by readers outside the State who know that by vote of the peoplé, some years ago, the Medical Department of the State University was located at Galveston. The finances of the University,—although the fund is ample,—are not yet in shape and available for the inauguration of a medical department; at least not in the full estate ; and according to the report of the Hon. President of the Board of Regents to the 20th Legislature, it is likely that the requisite amount of money will not be available for the full equipment and opening of the medical department for several years; but it has been suggested by those who are in a position to know, that a beginning might be made with the funds at hand, and that a good beginning might be the employment of a portion of the funds available, for the purpose indicated above, to-wit: the founding of certain branches—which could be operated in connection with the Galveston School—though under separate management—a *preparatory* school, as it were, of the University—and thus a cooperation would be established to a common end, until the Regents of the University shall become ready to assume control of the whole. It is understood that when the University funds are sufficient, and the time has arrived for opening the medical department, all the property of the Texas Medical College is to be turned over to them. Then, the present undertaking is the germ of the Medical Department of the University of Texas. Of course, at the metamorphosis a new organization will be effected.

It may not be out of place here to allude to the fact that the act creating a Medical Department of the University provides for *free medical education!* It seems to us that the sons of Texas have no claim on the State for free professional education, and that it is a mistake. Education in medicine, as before stated, is so cheap that it has well nigh brought the very name into disrepute ; and to give it away, as is proposed by this act, will surely defeat the cherished idea and imperative necessity of a higher standard. The curriculum of the Medical Department of the University of Texas should be as high and as complete, the instruction as thorough, as in any school in America, and the teachers as able ; moreover, the fees should be as high as the highest, and there should be a three year's graded course, with a preliminary examination for matriculation, The exigency of the times and circumstances demand all this, and the profession of Texas should be satisfied with nothing less.

Let the gentlemen in immediate charge remember that their success is impossible without the hearty good will and cooperation of the profession of the State, and this cooperation and support will not be accorded to any half-way measures ; to nothing short of an evident and earnest desire to make the school an honor and a credit to them, and to the great State ofTexas ; this fact should be a constant spur and incentive, and their success is certain.

